# Enhancement of soybean nodulation by seed treatment with non–thermal plasmas

**DOI:** 10.1038/s41598-020-61913-3

**Published:** 2020-03-18

**Authors:** María Cecilia Pérez-Pizá, Ezequiel Cejas, Carla Zilli, Leandro Prevosto, Beatriz Mancinelli, Diego Santa-Cruz, Gustavo Yannarelli, Karina Balestrasse

**Affiliations:** 1 Instituto de Investigaciones en Biociencias Agrícolas y Ambientales (INBA), Facultad de Agronomía, Universidad de Buenos Aires (UBA), Consejo Nacional de Investigaciones Científicas y Técnicas (CONICET), San Martín, 4453 Buenos Aires, Argentina; 2 Universidad Tecnológica Nacional, CONICET, Facultad Regional Venado Tuerto, Departamento de Ingeniería Electromecánica, Grupo de Descargas Eléctricas, Laprida 651, Venado Tuerto, Santa Fe, Argentina; 3 Laboratorio de Regulación Génica y Células Madre, Instituto de Medicina Traslacional, Trasplante y Bioingeniería (IMeTTyB), Universidad Favaloro-CONICET, Buenos Aires, Argentina

**Keywords:** Rhizobial symbiosis, Plasma physics

## Abstract

Soybean (*Glycine max* (L.) Merrill) is one of the most important crops worldwide providing dietary protein and vegetable oil. Most of the nitrogen required by the crop is supplied through biological N_2_ fixation. Non-thermal plasma is a fast, economical, and environmental-friendly technology that can improve seed quality, plant growth, and crop yield. Soybean seeds were exposed to a dielectric barrier discharge plasma operating at atmospheric pressure air with superimposed flows of O_2_ or N_2_ as carrying gases. An arrangement of a thin phenolic sheet covered by polyester films was employed as an insulating barrier. We focused on the ability of plasma to improve soybean nodulation and biological nitrogen fixation. The total number of nodules and their weight were significantly higher in plants grown from treated seeds than in control. Plasma treatments incremented 1.6 fold the nitrogenase activity in nodules, while leghaemoglobin content was increased two times, indicating that nodules were fixing nitrogen more actively than control. Accordingly, the nitrogen content in nodules and the aerial part of plants increased by 64% and 23%, respectively. Our results were supported by biometrical parameters. The results suggested that different mechanisms are involved in soybean nodulation improvement. Therefore, the root contents of isoflavonoids, glutathione, auxin and cytokinin, and expansin (GmEXP1) gene expression were determined. We consider this emerging technology is a suitable pre-sowing seed treatment.

## Introduction

Soybean (*Glycine max* (L.) Merrill) crop is considered a highly valuable source of protein, oil, and biofuel. With a worldwide sown area of 130 million hectares, soybean production achieved 370 million metric tons (68% of global production) in 2018–2019, up 9 percent from the previous year. Approximately 45% of this production corresponds to South American countries: Argentina, Brazil, and Paraguay^1^.

Growers in the main agricultural areas of the world employ chemical fertilizers as the only reliable tool for facing the increasing demand for food that accompanies the ever-growing world population^2^. There is a substantial concern around the world for the optimization of alternative nitrogen sources that may help to avoid the intensification of chemical fertilizer usage. The contribution of biological nitrogen fixation (BNF) to the stock of N available for the crop results essential. The symbiotic associations between legumes and rhizobia represent the most important N_2_-fixing agents in agricultural systems. Soybean is considered among the most efficient legume species regarding the ability to fix N: 58–68% of soybean plant N derives from N_2_ fixation^3^.

Only bacteria contain the nitrogenase enzyme that can reduce N_2_ to ammonium, and the major problem in maintaining a high rate of N_2_ fixation is that this enzyme is oxygen-labile^4^. Leghaemoglobin accumulated in the cytoplasm of infected plant cells binds to free oxygen avoiding the inactivation of nitrogenase^5^. Many scientific efforts have been made in the last decades to develop different strategies for the enhancement and exploitation of BFN potential: the breeding of legumes for N_2_-fixation, rhizobial strain selection, development of inoculants and amendments, adoption of determined agronomical management practices, among others. According to Giller & Cadisch^6^, the proper implementation of the already available agronomic knowledge plus the employment of simple technology could dramatically increase BNF, in shorter-terms than any other possible strategy.

Non-thermal plasmas (NTP) or cold atmospheric plasmas are a novel technology that is currently being studied worldwide, at laboratory scale^7^. Dry, non-thermal and rapid processes, characterized for leaving no environmental residues after their employment, are used to generate the NTP. Dielectric barrier discharge (DBD) is a novel method of NTP generation with low power requirements and excellent treatment flexibility, so a wide range of biological targets can be addressed^8^. In our previous investigations^9,10^ we demonstrated that NTP were suitable for seed treatment as positive effects were observed on the quality of seeds after treating them, besides the treatments resulted harmful for the fungal cells that were infecting the seeds. Moreover, we investigated the mechanism underlying the positive effects of plasma treatments on seed quality and the long-term effects on plant growth when applied to soybean seeds of different health states. It was proposed that changes in wettability-hydrophilicity of seed coats and hormonal profile, mediated by plasma-produced reactive oxygen species (ROS), were playing a key role in the enhancement of seed germination and the improvement of plant growth. It was also demonstrated that plants grown from plasma-treated seeds were superior in terms of growth and agronomic traits compared to the ones grown from non-treated seeds. The mechanisms behind this ultimate behavior remained unclear, besides it was suggested that it could be a consequence of the enhancement of seed quality. According to Ling *et al*.^11^ plasma can enhance enzyme digestion and mobilization of storage substances, thus increasing the concentrations of soluble sugar and soluble protein, thereby improving the germination rates and early seedling growth. Recently, Zhang *et al*.^12^ found that plasma could positively affect soybean sprouts growth by regulating the expression of certain growth-genes. According to Perrot-Rechenmann^13^, who sustained the “acid growth theory”, cellular expansion begins when auxin activates the plasma membrane H^+^-ATPases subsequently releasing protons into the apoplast, which acidifies the extracellular matrix. This acidification activates the loss of cell wall components network through triggering the action of expansins, which together with water uptake into the cell, provides the required turgor pressure that causes cell enlargement. Expansins are a group of proteins considered the main agents for cell wall elongation. According to Cosgrove^14^, they act affecting the stability of hydrogen bonds that unite cellulose and hemicellulose microfibrils provoking cell wall loosen and allowing the subsequent turgor-driven cell expansion, which irreversibly changes cell size and shape. GmEXP1 is a soybean root-specific expansin gene belong to the α-expansins, a group of genes known to be involved in root development. Lee *et al*.^15^ demonstrated that the over-expression of GmEXP1 in transgenic tobacco (*Nicotiana tabacum*) plants caused accelerated root growth.

Hayashi *et al*.^16^ proposed that plant growth promotion by plasma could be explained by the enhanced production of reduction-type thiols (thioredoxin and glutathione) and polyphenols, triggered by the produced ROS. Interestingly, these thiols are critical molecules in nodulation as well. GSH is believed to participate in morphogenesis, cell division, and control of redox status of cells, processes that are important in nodule formation and functioning^17^ while thioredoxins are essential to lower ROS levels during nodulation, contributing to nodule development and maintenance of the symbiotic state^18^. Polyphenols are a large class of chemical compounds that includes flavonoids, a diverse class of secondary plant metabolites known to play an essential role in promoting N_2_ fixing symbiosis with rhizobia^19^. The isoflavones daidzein and genistein, have been identified as the major molecules inducing nod genes of *Bradyrhizobium japonicum* (the specific rhizobia for soybean) which expression leads to the production and excretion of lipo‐oligosaccharides that elicit nodulation‐related responses in plants^20^.

In the light of this knowledge, the main objective of the present study was to investigate the possible mechanisms involved in nodular development and the process of biological nitrogen fixation in soybean plants grown from seeds treated with NTP. This research will enable us to thrust forward in clarifying the mechanisms underlying the long-term effects of this technology.

## Results

### Early responses of seeds and seedlings to plasma treatments

**Figure 1:**
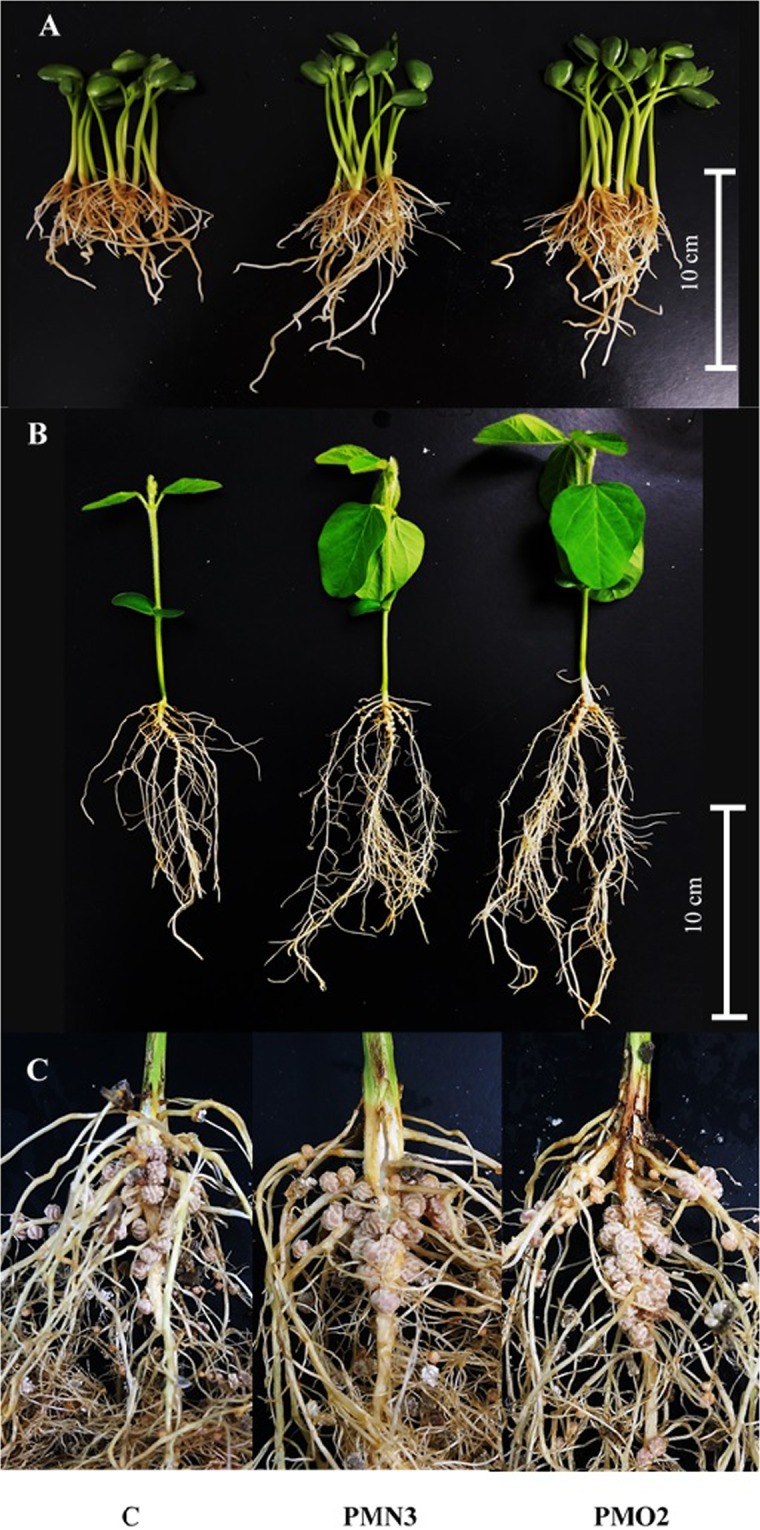
General aspect of (**A**) 5-d-old seedlings, (**B**) 15-d-old plants and (**C**) roots and nodules, grown from plasma treated seeds (PMN3 and PMO2), as well the control.

**Figure 2:**
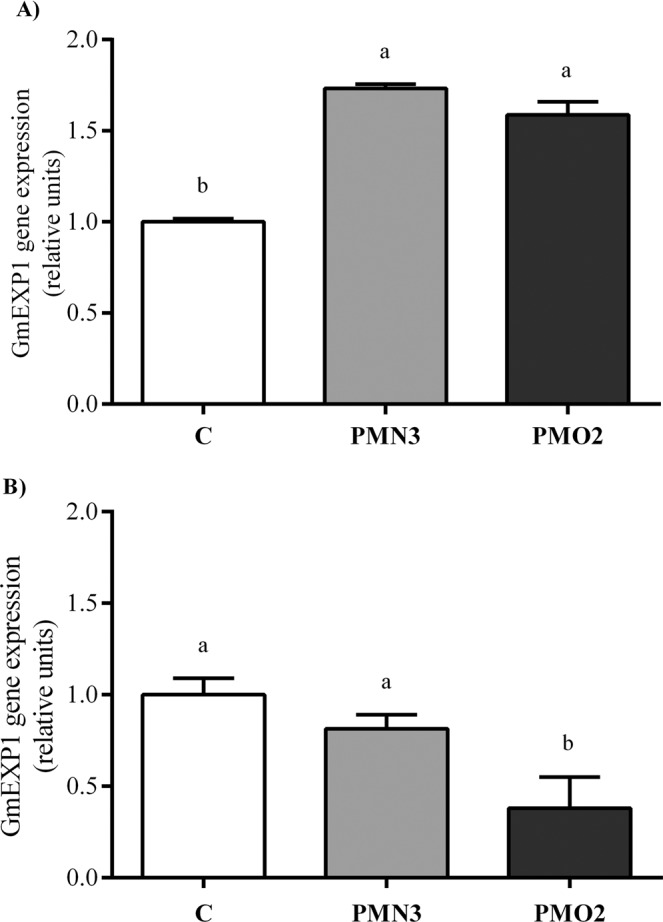
GmEXP1 gene expression in roots of (**A**) 5-d and (**B**) 15-d of plants grown from plasma treated seeds (PMN3 and PMO2) respect to control, which was expressed as 1 unit. Error bars indicate standard error (n = 3). Different lowercase letters denote statistical differences between groups (Tukey´s HSD test, P < 0.05).

Water absorption curves (see Supplementary Fig. S1) showed a significant influence of plasma treatments on seed water consumption during imbibition. Table 1 shows protein and soluble carbohydrates content in 5-d-old seedlings. Seeds treated with plasma showed significantly lower amounts of protein (8% on average) and higher levels of soluble carbohydrates (11% on average) with respect to the control (C). On the other hand, the length of 5-d-old seedlings (Table 1) revealed great and significant differences between seedlings grown from treated and from non-treated seeds, being 1.2 fold higher in treatments than in control. These differences responded mainly to enhanced root growth in response to plasma treatment (Fig. 1A), a phenomenon that we had already reported in our previous research^10^. The expression of GmEXP1 gene in roots (Fig. 2), revealed a 1.7-fold increase in both plasma treatments respect to control.

**Table 1:** Total protein content, soluble carbohydrates content and length of 5-d-old seedlings grown from plasma treated seeds (PMN3 and PMO2) and from non-treated seeds (C).

Treatment	Total protein	Soluble carbohydrates	Length
	(mg g fw^−1^)	(mg dL^−1^)	(cm)
C	37.6 ± 0.2a	14.0 ± 0.5a	15.5 ± 0.0b
PMN3	35.2 ± 0.2b	15.3 ± 0.4b	17.8 ± 0.1a
PMO2	34.3 ± 0.2b	15.7 ± 0.6b	18.7 ± 0.0a

Data show mean values of ten replicates ± standard error. Different lowercase letters denote statistical differences between the groups (Tukey´s HSD test, P < 0.05).

### Long-term effects of plasma treatments: early stages of vegetative growth

**Figure 3:**
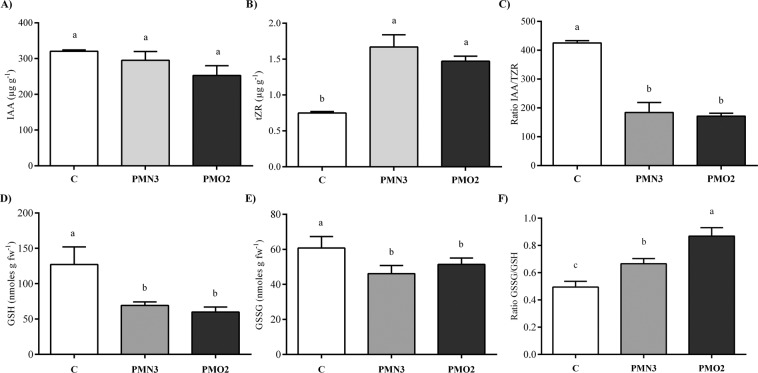
Phytohormones (IAA and tZR) and glutathione (GSH and GSSG) contents in roots of 15-d-old plants grown from plasma treated seeds (PMN3 and PMO2), as well the control. (**A**) IAA, (**B**) tZR, (**C**) IAA/tZR ratio, (**D**) GSH, (**E**) GSSG and (**F**) reduced/oxidized (GSSG/GSH) ratio. Error bars indicate standard error (n = 5), different lowercase letters denote statistical differences between groups (Tukey´s HSD test, P < 0.05).

At 15-d-old stage, significant differences were found between plants grown from treated seeds and controls concerning fresh weight and length (see Supplementary Table S2). Total fresh weight of plasma treatments (PMN3 and PMO2) were 1.2 folds incremented in comparison with control (C). These differences responded to increments in radical (22–28%) and aerial (18–20%) fresh weights. Total length was significantly augmented (4–10%) in plants grown from treated seeds in comparison with controls (Fig. 1B), and this behavior was related mainly to changes in root length (see Supplementary Table S2). By contrast, the aerial length of plasma treatments equalled control. Isoflavonoid content in roots of 15-d-old plants (see Supplementary Table S2) showed differences between plasma treatments and control. Daidzein, Genistein, and Daidzin were 1.5–1.8 folds diminished in plasma treatments with respect to control, while no differences were detected for Genistin content (see Supplementary Fig. S2). On the other hand, phytohormone levels in roots showed no differences between treatments and control for IAA (Fig. 3A), although there may be observed a tendency towards an increase in the control situation. The levels of tZR (Fig. 3B) correlated negatively with IAA content, showing increases of 1.9–2.2 fold for plasma treatments respect to control, nevertheless IAA/tZR ratio (Fig. 3C) diminished 2.3–2.6 times. Total glutathione content in roots evidenced a strong diminution 1.6-fold) in plasma treatments respect to control, due mainly to decreases of about 50% in GSH content. By contrast, the GSSG content did not vary (Fig. 3E), and the GSSG/GSH ratio (Fig. 3F) was 1.3–1.8 fold higher in treatments than in control. The expression of GmEXP1in roots (Fig. 2) exhibited an 11-fold and 23-fold decrease in control and treatments (respectively) in comparison with the 5-d-old control gene expression. Plasma treatments evidenced 1.2-fold and 2.6-fold decrease GmEXP1 expression with respect to 15-d-old control (Fig. 2).

### Long-term effects of plasma treatments: nodulation traits

**Figure 4:**
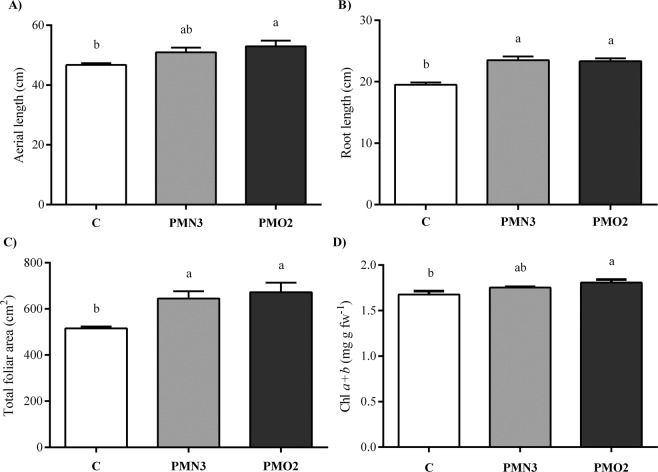
Biometrical parameters of 40-d-old plants grown from plasma treated seeds (PMN3 and PMO2) and from non-treated seeds (control). (**A**) Aerial and (**B**) radical lengths, (**C**) total foliar area and (**D**) total chlorophyll content in leaves. Error bars indicate standard error (n = 10). Different lowercase letters denote statistical differences between groups (Tukey´s HSD test, P < 0.05).

**Figure 5:**
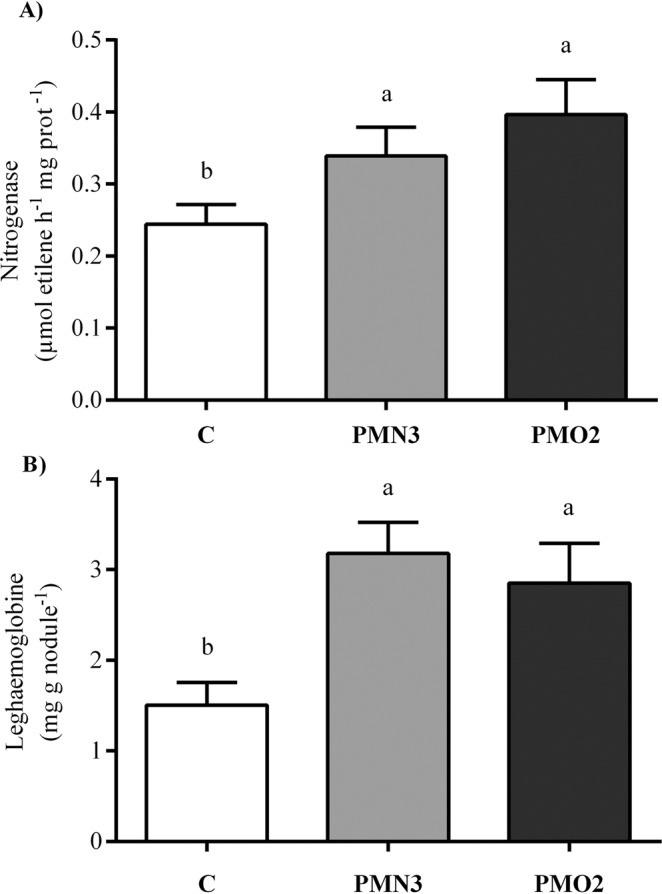
Nitrogenase activity and leghaemoglobin content in nodules of 40-d-old plants grown from plasma treated seeds (PMN3 and PMO2), as well the control. Error bars indicate standard error (n = 5). Different lowercase letters denote statistical differences between groups (Tukey´s HSD test, P < 0.05).

Long term effects of plasma treatment on 40-d-old plant biometry, are presented in Fig. 4. An enhancement of aerial length of about 12% was evidenced at this stage of growth (Fig. 4A), an effect that was detected at 5-d-old stage but then attenuated at 15-d-old. Coinciding with the observations made at the 5-d-old and 15-d-old stages, plasma treatments led to a 1.2-fold enhancement of root length, compared with the control (Fig. 4B). Total foliar area (Fig. 4C) and total chlorophyll content in leaves (Fig. 4D) were enhanced by plasma treatment, showing increments of 25–30% and 5–10% (respectively) in comparison with the control. On the other hand, total nodule biomass (Fig. 1C, Table 2) was significantly influenced by plasma. Plasma treatments elevated the average number of nodules in the primary root from 17 to 20–21, also showing an enhancement effect on fresh weight of total nodules, which weighted 73% (on average) more than control (Table 2). Likewise, individual nodules were heavier than control, showing increments of 48% and 72% in fresh and dry weights, respectively (Table 2). Nitrogenase activity and leghaemoglobin content in nodules of 40-d-old plants are shown in Fig. 5, where strong effects of plasma treatments on both parameters can be easily observed. Plants grown from treated seeds evidenced a nitrogenase activity in nodules 1.4–1.6-fold higher than control (Fig. 5A), while the leghaemoglobin content increased twice (Fig. 5B). On the other hand, Table 3 presents the values of total N-content per plant, discriminated according to the type of tissue (aerial, radical, and nodular). Plasma treatments evidenced increments of 25% in the total content of N per plant. N-content in the aerial part of plants augmented 40% (on average) respect to control, while the N-content in nodular tissues was 2.1–3 folds higher. By contrast, the content of N in roots grown from treated seeds was 28% (on average) lower than control.

**Table 2:** Nodulation traits in 40-d-old plants grown from plasma treated seeds (PMN3 and PMO2), as well the control: number and fresh weight of total nodules in the main root, and dry matter and water contents per nodule.

Treatment	Nodules in the main root				
	Number	Total fw	Fw per nodule	Total dw	Dw per nodule
		(g)	(mg)	(g)	(mg)
C	17 ± 0.2b	1.0 ± 0.1b	58.4 ± 3.1c	0.19 ± 0.01c	10.9 ± 0.3c
PMN3	20 ± 0.1a	1.6 ± 0.1a	80.4 ± 1.2b	0.29 ± 0.01b	14.4 ± 0.3b
PMO2	21 ± 0.3a	1.9 ± 0.1a	91.8 ± 1.0a	0.36 ± 0.02a	17.1 ± 0.5a

Data show mean values of ten replicates ± standard error. Different lowercase letters denote statistical differences between the groups (Tukey´s HSD test, P < 0.05).

**Table 3:** Total and partitioned nitrogen content of 40-d-old plants grown from plasma treated seeds (PMN3 and PMO2), as well the control.

Treatment	Nitrogen content per plant (mg)			
	Total	Stem and leaves	Root	Nodules
C	223.18 ± 4.08c	150.74 ± 8.82b	61.60 ± 5.34a	10.80 ± 0.45c
PMN3	270.50 ± 8.06b	206.31 ± 7.74a	41.86 ± 1.52b	22.33 ± 3.16b
PMO2	294.60 ± 7.35a	215.97 ± 6.38a	46.59 ± 0.75b	32.04 ± 1.62a

Data show mean values of ten replicates ± standard error. Different lowercase letters denote statistical differences between the groups (Tukey´s HSD test, P < 0.05).

## Discussion

Seed germination begins with the absorption of water by the dry seed and culminates with the emergence of the radicle; throughout imbibition, the seed passes from a quiescent state to a state of highly active metabolism^21^. The imbibition curves obtained in this study (see Supplementary Fig. S1) evidenced significantly enhanced water absorption in treated seeds respect to control. During germination and early growth, reserve proteins in cotyledons are digested by the action of proteases to obtain amino acids and energy necessary to sustain seedling growth. Consequently, protein content in the seed decreases gradually during imbibition as a natural process of germination, known to be mediated by regulated proteolysis rather than by *de novo* synthesis of proteins^21^. The protein contents determined in the present work (Table 1) were lower in treated than in non-treated seeds, contrary to what happened with soluble carbohydrates content (Table 1) which correlated positively with the stimulation of water absorption (see Supplementary Fig. S1) and the subsequent enhanced early growth (Table 1). In our latest research^10^, we demonstrated that seeds treated with plasma (PMN3 and PMO2 treatments) germinated better, faster, and more vigorously than the non-treated ones. Altogether, our observations suggest that plasma treatments provoke a substantial acceleration of the germination process, which begins with enhanced water absorption and is followed by stimulated digestion and mobilization of reserve compounds present in the seed, as it was proposed by Ling *et al*.^11^. The length of 5-d-old seedlings (Fig. 1A) showed that seed exposition to plasma treatment resulted in enhanced seedling growth. As we revealed in our previous work^10^, the earlier response to plasma treatments that may be macroscopically observed in seedlings is the enhanced root growth. This advantage upon control may have propitiated improvements in the ability of plants to explore the soil and to capture and uptake water and nutrients^22^. Moreover, considering that the tips of emerging root hairs are the primary targets for infection by rhizobia^23^, it can be thought that seedling grown from plasma treatments had more opportunities than control to stablish relations with rhizobia, as their roots were developing faster and more vigorously.

Coinciding with the results showed for 5-d-old seedlings, the biometrical parameters at 15-d-old (Fig. 1B) evidenced strong differences between plants grown from plasma treatment seeds and control, mainly in terms of total fresh weight, radical fresh weight, and root length (see Supplementary Table S2). Regarding the aerial part, the fresh weight of plants grown from treated seeds was higher than control, but stem length had no difference (see Supplementary Table S2), meaning that the increment in fresh weight was probably related to changes in others aerial parameters rather than in stem length. This suggestion is in accordance with Ling *et al*.^11^, who reported markedly raised leaf size (area and thickness) and stem diameter in peanut plants grown from plasma-treated seeds. On the other hand, we observed at this stage striking visual differences between plasma treatments and control, concerning plant nodulation (Fig. 1B). To understand further the mechanism by which plasma improved root growth, we investigated the expression profile of the soybean-root-specific expansin gene, GmEXP1 (Fig. 2). This gene is known to express mostly when primary and secondary root elongations take place, with a maximum of expression at 5-d after germination^15^. Its overexpression leads to cell elongation and accelerated root growth. Our results showed that roots grown from plasma treatments expressed the GmEXP1 gene to a significantly greater extent than control, at the 5-d-old stage^15^. According to cell growth theories^13,24^, cellular expansion is the result of the concatenated action of auxin, expansin, and cell/vacuole turgor. Our results demonstrated that the enhanced root size observed in PMN3 and PMO2 at 5-d-old stage, correlated positively with GmEXP1 gene expression. As the expression pattern of this expansin gene is known to be temporally regulated during root development^15^, a natural decrease in GmEXP1 gene expression was expected. When comparing treatments with 15-d-old control, we observed a markedly decrease in treatments gene expression, meaning that roots were transiting different phases of root development and that treatments where advanced. This suggestion is supported by the differential root growth showed in Fig. 1B and agrees with what we demonstrated in our previous study^10^.

To elucidate the mechanism involved in the differential nodulation between plants growing from treated and non-treated seeds, some parameters known to be involved in root cell growth and nodule formation (flavonoid production, phytohormones profile, tiol content) were determined. The symbiotic interaction and consequent nodule formation is initiated by the host plant roots exuding phenolic compounds into the rhizosphere, mainly flavonoids and isoflavonoids^25^. Our results showed a significant decrease in daidzein, genistein, and daidzin contents in roots belonged to plasma treatments (see Supplementary Fig. S2). According to Ferguson^26^, nodule formation follows a highly ordered and coordinated chain of steps that ends in the formation of a N_2_ fixing mature nodule. In Fig. 1B, it can be appreciated that plants grown from treated seeds were transiting a differential and advanced stage in nodule formation, with respect to control. The reduction in isoflavonoids contents observed in plasma treatments might be explained by this advancement in the nodulation process: while control roots are in an earlier stage of nodule formation, where isoflavonoids production is needed, roots that belong to plasma treatments no longer produce those components because they have already abandoned that phase. It is well known that nodule initiation and development are mediated by phytohormones, mainly IAA and cytokinins^26,27^. Gresshoff^28^ proposed the ABC (auxin-burst-control) hypothesis for autoregulation of nodules formation, which sustains that once a determined amount of nodule initiation has occurred, the shoot of the plant responds through an increase in translocation of auxins, leading to an auxin burst in roots, which results inhibitory to further nodule initiation. In addition to auxin, cytokinins are required for cell division at a certain ratio to auxin^27^. In the present work, the levels of IAA (Fig. 3A) and tZR (Fig. 3B) in roots of plants grown from treated seeds were determined and compared with the non-treated control. Our results denoted that auxin levels were present at higher concentrations in control roots, although no significant differences could be detected with respect to plasma treatments. By the contrary, tZR contents were higher in roots belonged to plasma treatments. These quantitative differences in root’s endogenous hormone status between treated and non-treated conditions might be related to the advanced nodulation. Nonetheless, the contrasting behavior of phytohormones in roots grown from treated seeds with respect to control reminded us to certain studies^27,29^ reporting changes in phytohormones profile of super or hyper-nodulating mutants of *Medicago truncatula* and soybean in respect to the wild types. According to the authors of these works, super-nodulating plants exhibited an alteration in the ABC mechanism: contained lower levels of auxin than the wild type because they were unable to generate the auxin burst in response to the first nodules formed. Specifically, soybean hyper-nodulating mutants were found to exhibit an auxin:cytokinin balance lower than the wild type, suggesting that this ratio was playing an important role in nodule number regulation^27^. Altogether, it is likely that the differences observed in our work with respect to IAA (Fig. 3A) and IAA/tZR ratio (Fig. 3C) may be explained by the alteration in the ABC mechanism, just like proposed for super-nodulating soybean mutants. Nonetheless, super-nodulating plants with altered ABC mechanisms are known to develop stunted and yield poorly^30^. This phenomenon was not observed in our work, as plants grown from plasma treated seeds showed improved growth and yield than control^10^. We suggest that if an alteration in the ABC mechanism took place in treatments, it must have been a slight one. In regard with thiols, Hayashi *et al*.^16^ observed that thioredoxin and glutathione production was involved in the promotion phenomenon induced by plasma, suggesting that the decrease in excess oxidative substances in a plant could boost its growth. Accordingly, we determined the quantity of GSH (Fig. 3D) and GSSG (Fig. 3E) in roots of 15-d-old plants, intending to clarify if the behavior of these thiols could help us explained the exacerbated plant growth. In our results, plasma treatments showed a GSH content greatly lower than control, while GSSG content did not vary. According to Dalton *et al*.^31^ oxidative stress occurs naturally in nodules when they produce H_2_O_2_ during ureides formation (the major transport forms of N from nodules in soybean). Roots nodules activate, in these cases, the ascorbate-GSH cycle, which requires a continuous supply of GSH to prevent N_2_ fixation mechanism from oxidation^32,33^. The GSSG/GSH ratio was demonstrated to slowly increase during the nodule development; while in all cases, the percentage of the oxidized form remained small^34^. Control plants showed the expected behavior concerning isoflavonoids and glutathione contents in roots, which confirms that both components were involved in nodulation at 15-d-old stage. The results obtained for plasma treatments reflect nothing else but an acceleration in the nodulation process respect to control.

Coinciding with the observations made at 5 and 15-d-old stages, plasma treatments enhanced plant length at the 40-d-old stage and this effect responded mainly to enhanced root growth (Fig. 4B). Our results also demonstrated that plasma treatments enhanced the total foliar area per plant (Fig. 4C) and total chlorophyll content in leaves (Fig. 4D). These parameters correlated positively with aerial (Fig. 4A) and radical lengths (Fig. 4B). Biomass production by a crop depends on the capacity of plants to intercept incident radiation, between other important factors^35^. In this sense, the more radiation is intercepted, the higher plant growth rate will be. According to Soundararajan^33^, there is a strong positive relationship between leaf chlorophyll content and carbon assimilation in soybean. This knowledge allows us to suggest that the improvement effect of plasma regarding foliar area and chlorophyll content could help us explaining the enhanced vegetative growth evidenced by plants grown from treated seeds. Our results are consistent with Kriz *et al*.^36^ who reported that plants grown from plasma treated seeds were more robust, had a stronger root system, and developed quicker than controls.

Concerning nodulation traits, in our work we observed that plasma treatments induced increments in nodule biomass (Fig. 1C, Table 2), total number of nodules (in the primary root), and water and dry matter contents (Table 2). It is well known that the number of nodules present on the root system is directly related to the amount of N_2_ fixation: a higher number of nodules have more N_2_ fixation potential^37^. Accordingly, N_2_ fixation, measured as nitrogenase activity, and leghaemoglobin content in nodules (Fig. 5A) were greatly improved by plasma treatments. These parameters correlated positively with N-content in the aerial part of plants (stem + leaves) and nodules, and negatively with its content in roots (Table 3). According to Herridge *et al*.^3^ nitrogenase activity determine the efficiency of nodules in fixing N_2_ while leghaemoglobin content denotes the efficiency of nodules in buffering free oxygen and avoiding the enzyme inactivation^5^. The N-content in aerial and nodular tissues was significantly incremented by plasma treatment and contributed to the enhanced average value of total N-content per plant. These results, supported by nitrogenase activity (Fig. 5B) and leghaemoglobin content in nodules (Fig. 5B), indicated that plasma treatments enhanced significantly the BNF. The contents of N in roots of plants grown from treated seeds were 24–32% lower than control, suggesting that the N fixed by nodules had been exported to the aerial part of plants by the moment of measurement. Warembourg & Fernandez^38^, who revealed that during soybean vegetative growth 47% of the N fixed by nodules is directed towards the leaves while only 14% remains in roots, supports this suggestion. It is well known that N is strongly linked to crop yield, since the amount of N accumulated in leaves determines the intensity of photosynthetic process^39^. Consequently, an increment in the availability of this nutrient may lead to higher carbon assimilation and subsequent grain yield. Moreover, N-fixation supplies approximately 90% of seed N^40^. In our previous study^10^, we demonstrated that plasma treatment (PMN3) was capable of enhancing soybean yield in terms of the number of pods (8%) and seeds (4%) per plant, total dry weight of seeds (11%) and weight of 1000 seeds (2%). The higher availability of nitrogen due to greater BNF observed in the present work could help us explaining this phenomenon. According to Deaker *et al*.^41^, regarding seed inoculation with rhizobia, factors such as desiccation may affect rhizobia survival on seed and applying protectant additives in the seed coating may ameliorate this adverse effect. Inoculant producers worry about increasing the number of bacteria per seed reaching the soil. Therefore they usually work developing different additives (protectors, adhesives) that help to improve bacterial survival and its adherence to seed^42^. Many studies^43–45^ have been carried out employing plasma technology to change the hydrophilic/hydrophobic properties of different materials. In our previous study^9^ we demonstrated that seed coats experimented increments in their hydrophilicity following the exposition to plasma atmosphere. These changes allowed seeds to improve water absorption during imbibition, which resulted positive for germination. Altogether, we suggest that the surface modification of seeds may have played an essential role in the enhancing of plant-rhizobia relation by propitiating inoculant adherence to seed. Recently, Strejckova *et al*.^46^ demonstrated that plasma technology modified seed surface properties, thus ensuring a good and uniform adhesion of beneficial fungi spores, which enhanced the biological value of seeds. This knowledge supports our suggestion about the beneficial effect of plasma regarding seed coating with rhizobia.

In conclusion, the earlier response of plants to plasma treatment was the increment in root growth. This advantage upon control may have propitiated improvements in later nodule formation, as root systems had greater ability to explore the soil and to capture and uptake water and nutrients. Then, the enhanced nodulation may have been responsible for the subsequent plant growth enhancement. Thus, the promotional effect of plasma on root growth might have been implicated in nodulation enhancement, and *vice versa*. To our knowledge, these long-term effects of plasma treatments on nodulation have not so far been reported neither for soybean nor for any vegetal species. As cold plasma technology can significantly improve the BNF, we propose it as a routine practice for seed treatment before sowing, not only for commercial but also for cover crops. By reducing the need for agrochemicals (fertilizers) during the growth cycle and improving the N contribution to the N stock in soils, the implementation of this practice will improve crop profitability and help to preserve the environment.

## Materials and Methods

### Plant material and non-thermal plasma treatments

DM 53i53 IPRO soybean seeds (Don Mario Semillas S.A., Buenos Aires, Argentina) were employed for guiding the experiments. Non-thermal plasma treatments were performed on these seeds as previously reported on Pérez Pizá *et al*.^9^. Plasma discharge consisted in a needle-array power electrode and a plate ground electrode covered by a dielectric barrier of an arrangement of a thin phenolic sheet (Pertinax, 2 mm thick) and 2 polyester films (Mylar, 100 µm thick). The gap between the upper surface of the barrier and the tip of the needles was fixed to 10 mm during the experiment. The power supply was a high-voltage sine AC power supply (0–25 kV) operating at 50 Hz. The setup of the experimental apparatus is described in detail in Pérez Pizá *et al*.^9^. O_2_ and N_2_ were alternatively injected into the discharge active region as carrying gases (measured gas-flow rate of 6 NL min^−1^) and seeds were treated with plasma exposures of 2 or 3 minutes (depending on the carrying gas), constituting plasma treatments PMO2 and PMN3. Non-treated seeds where employed as control (C).

### Growing conditions

Control and plasma-treated seeds were subjected to the Between Paper (BP) germination method proposed by ISTA^47^ and placed in a growth chamber under temperatures of 24/25 °C. Seeds were let to germinate for 5 days. Seedlings were taken at random from each growing bed (five EU with five seedlings each) and subjected to determinations of total protein and soluble carbohydrate. Measures of length and fresh weight of seedlings were also performed in this instance. In parallel, control and plasma-treated seeds were sown, and plants were grown in sterilized vermiculite in 1-liter plastic pots and maintained in a growth chamber with a 16-h photoperiod and day/night temperatures of 24/25 °C, with a photosynthetic photon flux density of 350 µE m^−2^ s^−1^. Each pot constituted an experimental unit (EU) and included two plants (except the ones for nitrogenase activity determination, where each EU was constituted by one plant in a pot). The EUs were distributed randomly inside the chamber for assuring uniforms conditions. Before sowing, seeds were inoculated with 1 µl g^−1^ of Signum® (*Bradyrhizobium* sp). Plants were irrigated every day with Hoagland solution without nitrogen^48^. One group of plants (five EU per treatment) were grown for 15 days and samples were collected to analyze biometry, isoflavonoids production, glutathione, phytohormones contents and GmEXP1 gene expression. Another group (ten EU per treatment) was followed for 40 days with the purpose of evaluating plant biometry (size and foliar area), chlorophyll content in leaves, total nitrogen content and nodulation traits (number, fresh and dry weights of nodules, nitrogenase activity, leghaemoglobin, protein and total nitrogen content).

### Quantification of soluble carbohydrates and total protein

Roots (1.0 g) were grounded and homogenized with 1 mg polyvinylpyrrolidone (PVP) in 3 mL of 50 mM phosphate extraction buffer (pH 7.7) containing 0.5 mM EDTA and 0.5% (v/v) of Triton X-100. The homogenates were centrifuged for 30 minutes at 13000 g and 4 °C. The supernatant fraction was used for the determination of soluble carbohydrates content employing *Glicemia enzimática AA* (Wiener lab.) kit, according to the manufacturer’s procedure. Protein concentration in imbibed seeds and seedlings was evaluated by the method of Bradford^49^, employing bovine serum albumin as a standard.

### Phytohormones and isoflavonoids determinations

Plants for phytohormone analysis were carefully uprooted, rinsed with distilled water and dried with filter paper. Roots from the upper half of the root system, where more than 80% of nodules are located in a mature soybean plants^27^, were taken from each plant and mixed. This material was weighed and stored at −80 °C until used. Frozen roots samples were grounded in a mortar using liquid nitrogen, weighted (1 g) and employed for indole acetic acid (IAA) and trans-zeatin riboside (tZR) determination. The extraction, purification and quantification were performed according to Hoyerová *et al*.^50^ and Dobrev & Kamınek^51^, with modifications. IAA, and tZR content were analyzed by HPLC (Agilent 1100 Series). For isoflavonoids content, frozen roots samples were grounded in a mortar with liquid nitrogen, weighted (0.5 g) and employed for the quantification of daidzein (7,4′-dihydroxyisoflavone), genistein (5,7,4′- Trihydroxyisoflavone) and their respective glucosides, daidzin (7,4′-dihydroxyisoflavone-7-O-glucoside) and genistin (5,7,4′-trihydroxyisoflavone-7-O-glucoside), according to Zavala *et al*.^52^.

### Expansin gene expression: Real-time quantitative RT-PCR

Total RNA was isolated from roots samples (100 mg) using 1 ml of TRIzol™ Reagent (Invitrogen), according to the manufacturer’s procedure. The ARN was treated with RNase-free DNase I (Promega) and reverse transcribed into cDNA using random hexamers and M-MLV Superscript II RT (Invitrogen). Quantitative RT-PCR was performed using soybean specific primers (see Supplementary, Table S1) on a StepOne real-time PCR system (Applied Biosystems). The threshold cycle (Ct) values were normalized against the reference gene 18 S (see Supplementary, Table S1). Results were calculated using the Relative Quantification (ΔΔCt) method^53^ and presented as the fold change in gene expression normalized and relative to the non-treated control.

### Glutathione content

Non-protein thiols were extracted by homogenizing 0.3 g of roots in 3 mL of 0.1 N HCl (pH 2) with 1 g polyvinylpyrrolidone (PVP). Extracts were centrifuged at 10000 g for 10 min at 4 °C and the supernatants were used for analysis. Total glutathione content (GSH + GSSG) was determined in the homogenates by spectrophotometry at 412 nm, employing glutathione reductase (GR), 5,5′-dithiobis-(2-nitrobenzoic acid) (DTNB) and NADPH. Oxidized glutathione content (GSSG) was determined by the same method in the presence of 2-vinylpyridine. Reduced glutathione content (GSH) content was calculated from the difference between the total glutathione content and GSSG content^54^.

### Chlorophyll content

Leaves (0.5 g of fresh weight) were homogenized with 96% ethanol (1:30 w/v). Extracts were heated in a boiling bath until complete bleaching. The absorbance was measured in the supernatant at 665, 649, and 654 nm as described by Wintermans and de Mots^55^.

### Plant biometry

Aerial and radical lengths were measure employing a measurement tape. Seedling length was the sum of epicotyl, hypocotyl and primary root lengths measured to the nearest half centimeter. Plant material for dry weight was dried at 80 °C for 120 h. Fresh and dry weights were registered employing an analytical balance (0.001 g). The area of each trifoliate leaf was determined employing the non-destructive millimetre graph paper method^56^.

### Nodulation traits

Fresh and dry weights of total nodules were registered employing an analytical balance (0.001 g). Material for dry weight was dried at 80 °C for 120 h. The number of total nodules per plant was determined by counting the total nodules harvested from each EU and dividing by two.

### Nitrogenase activity and leghaemoglobin content

Nitrogen fixation was measured by the acetylene (C_2_H_2_) reduction assay^57^, based on the ability of enzyme nitrogenase to reduce acetylene (C_2_H_2_) to ethylene (C_2_H_4_). The root system was removed from each plant, washed carefully (with distilled water) and placed in a 650 mL flask. Each flask, containing C_2_H_2_ (10%, v/v) in air, was sealed with a screw cap and surrounded by cling film. After 4 hours, 1.0-mL gas sample was removed using a syringe and analysed for ethylene on a gas chromatograph (Khon 3000 HRGC). Ethylene was quantified by comparison of peak areas with those produced by known amounts of the hormone and normalized dividing the content by the protein content of the nodules in each flask. For leghaemoglobin quantification, nodules (0.3 g) were homogenised in 3 mL of extraction medium containing 0.02% (w/v) potassium (K) ferricyanide and 0.1% sodium bicarbonate. Leghaemoglobin content was measured in the supernatant (obtained after centrifugation at 3000 g), using a fluorometric method as described^58^ and expressed as mg g fw^−1^.

### Total nitrogen content

The vegetal material (leaves, stems, roots and nodules) for total nitrogen determination was dried at 80 °C for 120 h, weighted (40–100 mg) and subjected to a wet digestion employing the sulfuric acid-hydrogen peroxide method proposed by Parkinson and Allen^59^. Total nitrogen content was measured according to Baethgen & Alley^60^ method, based on the color reaction between ammonium and a weakly alkaline mixture of Na salicylate and a chlorine source (Na-hypochlorite 0.3%). The intensity of the green color formed (enhanced by the use of Na nitroprusside) was measured at 650 nm. Sodium tartrate was added to eliminate the precipitation of hydroxides of heavy metals which could be present in the digests. The concentration of ammonium (NH_4_-N) in the samples was expressed in μg N ml^−1^.

### Statistical analysis

The experiment was carried out in triplicate. Data, mean values of replicates, are representative of the three experiments. Analyses were performed using the statistic software package RCommander version 3.1.2 (2014). The variance (p < 0.05) of data was analyzed by one-way analysis of variance (ANOVA), after testing for the assumption of the normal distribution (Shapiro-Wilk´s test) and of homogeneity of variances (Levene’s test). Tukey’s HSD (Honestly-significant-difference) tests were performed for multiple comparisons of groups by means (p < 0.05).

## Supplementary information

Supplementary information.

## Data Availability

The datasets generated during the current study are available from the corresponding author on reasonable request.
