# Supplementary material for: Enhancement of soybean nodulation by seed treatment with non–thermal plasmas
Source: Sci Rep. 2020 Mar 18;10:4917. doi: 10.1038/s41598-020-61913-3 (PMC7080784; doi:10.1038/s41598-020-61913-3)
Supplement: Supplementary file 1 — Supplementary information. [file 41598_2020_61913_MOESM1_ESM.pdf]

Enhancement of soybean nodulation by seed treatment with non–thermal plasmas

María Cecilia Pérez-Pizá, Ezequiel Cejas, Carla Zilli, Leandro Prevosto, Beatriz Mancinelli, Santa-Cruz Diego, Gustavo Yannarelli, Karina Balestrasse.

Supplementary information

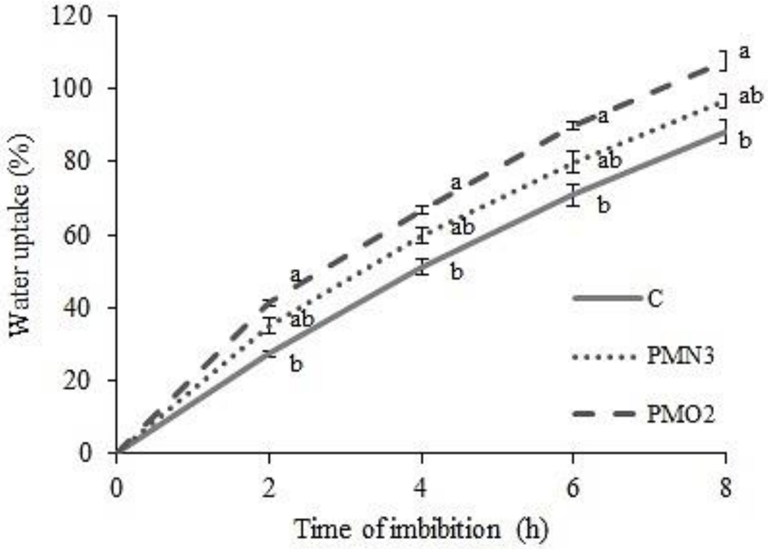

Figure S1. Water consumption during the first 8 hours of imbibition of plasma treated seeds, as well the control. Error bars indicate standard error (n = 5). Different lowercase letters denote statistical differences between groups (Tukey´s HSD test, P <0.05).

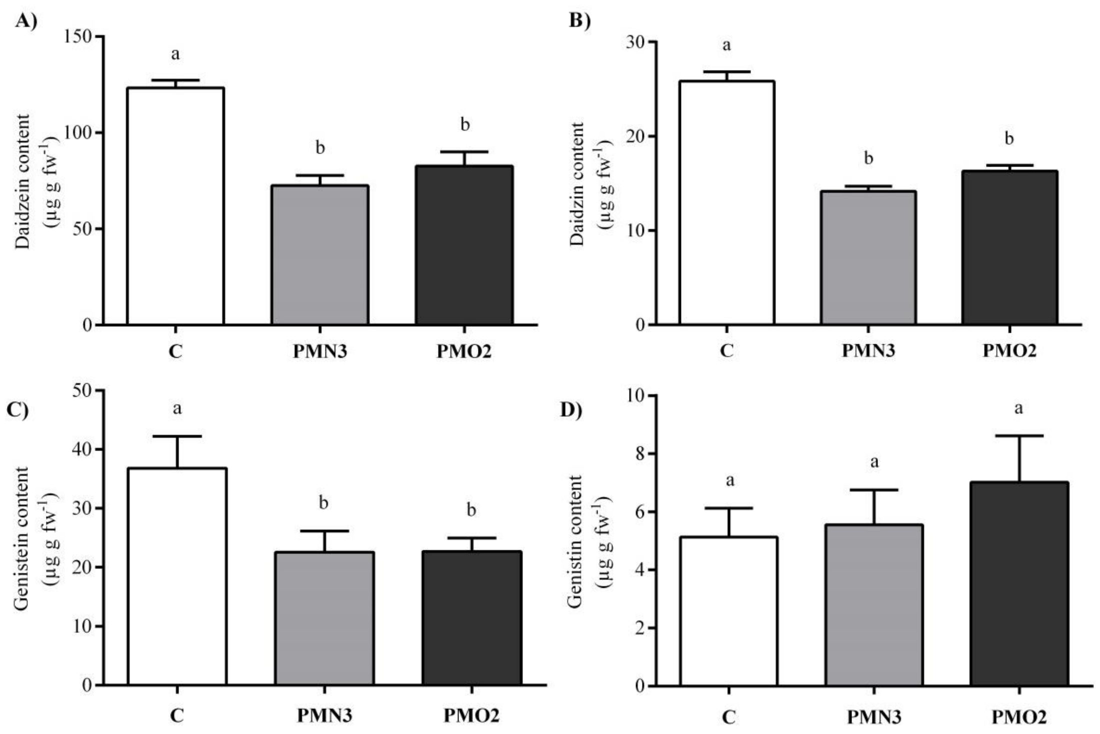

Figure S2. Isoflavonoids contents in roots of 15-d-old plants grown from plasma treated seeds (PMN3 and PMO2), as well as from the control. A) Daidzein, B) daidzin, C) genistein and D) genistin. Error bars indicate standard error (n = 5), different lowercase letters denote statistical differences between groups (Tukey´s HSD test, $P < 0.05$).

Table S1. Sequences of primers used for GmEXP1 gene expression.

Primer		Sequence
18S	Forward	5’-GGCTACCACATCCAAGGAA-3’
	Reverse	5’-CTATTGGAGCTGGAATTACCG-3’
GmEXP1	Forward	5’-GGATCGTCCCCGTGCTATT-3’
	Reverse	5’-GAACCTAACCCCTCCATGCTT-3’

Table S2. Total and partitioned (radical and aerial) fresh weights and lengths of 15-d-old soybean plants grown from plasma treated seeds (PMN3 and PMO2), as well the control.

Treatment	Fresh weight (g)			Length (g)		
	Total	Radical	Aerial	Total	Radical	Aerial
C	2.5 ± 0.1b	1.1 ± 0.1b	1.4 ± 0.1b	26.8 ± 1.1b	15.9 ± 0.8b	10.9 ± 0.4a
PMN3	3.0 ± 0.1a	1.4 ± 0.1a	1.7 ± 0.0a	27.9 ± 0.3ab	16.4 ± 0.4ab	11.5 ± 0.4a
PMO2	3.1 ± 0.1a	1.4 ± 0.1a	1.7 ± 0.1a	29.6 ± 0.3a	18.5 ± 0.5a	11.1 ± 0.6a

Data show mean values of five replicates ± standard error. Different lowercase letters denote statistical differences between the groups (Tukey´s HSD test, $P < 0.05$).
